# Molecular Dynamic Simulations Reveal the Structural Determinants of Fatty Acid Binding to Oxy-Myoglobin

**DOI:** 10.1371/journal.pone.0128496

**Published:** 2015-06-01

**Authors:** Sree V. Chintapalli, Gaurav Bhardwaj, Reema Patel, Natasha Shah, Randen L. Patterson, Damian B. van Rossum, Andriy Anishkin, Sean H. Adams

**Affiliations:** 1 Arkansas Children’s Nutrition Center, and Department of Pediatrics, University of Arkansas for Medical Sciences, Little Rock, Arkansas, United States of America; 2 Department of Biochemistry and Molecular Medicine, School of Medicine, University of California Davis, Davis, California, United States of America; 3 Department of Physiology and Membrane Biology, School of Medicine, University of California Davis, Davis, California, United States of America; 4 Center for Computational Proteomics, The Pennsylvania State University, State College, Pennsylvania, United States of America; 5 Department of Biology, The Pennsylvania State University, State College, Pennsylvania, United States of America; 6 Department of Biology, University of Maryland, College Park, Maryland, United States of America; Computational Biophysics, GERMANY

## Abstract

The mechanism(s) by which fatty acids are sequestered and transported in muscle have not been fully elucidated. A potential key player in this process is the protein myoglobin (Mb). Indeed, there is a catalogue of empirical evidence supporting direct interaction of globins with fatty acid metabolites; however, the binding pocket and regulation of the interaction remains to be established. In this study, we employed a computational strategy to elucidate the structural determinants of fatty acids (palmitic & oleic acid) binding to Mb. Sequence analysis and docking simulations with a horse (*Equus caballus*) structural Mb reference reveals a fatty acid-binding site in the hydrophobic cleft near the heme region in Mb. Both palmitic acid and oleic acid attain a “U” shaped structure similar to their conformation in pockets of other fatty acid-binding proteins. Specifically, we found that the carboxyl head group of palmitic acid coordinates with the amino group of Lys45, whereas the carboxyl group of oleic acid coordinates with both the amino groups of Lys45 and Lys63. The alkyl tails of both fatty acids are supported by surrounding hydrophobic residues Leu29, Leu32, Phe33, Phe43, Phe46, Val67, Val68 and Ile107. In the saturated palmitic acid, the hydrophobic tail moves freely and occasionally penetrates deeper inside the hydrophobic cleft, making additional contacts with Val28, Leu69, Leu72 and Ile111. Our simulations reveal a dynamic and stable binding pocket in which the oxygen molecule and heme group in Mb are required for additional hydrophobic interactions. Taken together, these findings support a mechanism in which Mb acts as a muscle transporter for fatty acid when it is in the oxygenated state and releases fatty acid when Mb converts to deoxygenated state.

## Introduction

Myoglobin (Mb) is a single polypeptide of ~153 amino acids expressed mainly in mammalian cardiac and skeletal tissues, and is also found in some invertebrate species [1–3]. Structurally, Mb is comprised of eight alpha helices (subdefined A-H), containing a heme (iron containing porphyrin) prosthetic group in the center and a hydrophobic core. Unlike tetrameric hemoglobin, Mb exists as a monomer and has a high affinity for oxygen, suggesting an important role in tissue O_2_ homeostasis and trafficking [3]. High concentrations of Mb in muscle cells may allow organisms to breath-hold longer as Mb can supply O_2_ during a transient decrease in the blood O_2_ levels, e.g. during the initiation of intense exercise or in deep diving marine mammals [4].

Besides this classically defined role, there is a growing body of literature showing that Mb impacts physiology beyond regulation of tissue O_2_ status. Mb disruption in mice resulted in no detectable impairment in cellular respiration and bioenergetics, but led to activation of multiple compensatory mechanisms [5–7]. It has been proposed that Mb protects mitochondrial respiration from bioactive nitric oxide by acting as an intracellular scavenger for this gaseous messenger, thus regulating its level in both the skeletal and cardiac muscle [8, 9]. Key to the present study, a role for Mb in fatty acid sequestration or trafficking is supported by extensive research.

The first experimental evidence of fatty acid-binding to globins was observed 30 years ago and the physiological linkage between globins and fatty acids has since been expanded. In seminal studies, ^14^C-labelled oleic acid was found to bind to Mb with an affinity 40 times lower compared to serum albumin [10]. In other experiments *Gotz et al*. determined that Mb has a higher binding affinity toward unsaturated fatty acids versus saturated fatty acids [11]. The Gotz *et al*. study also indicated that the oxygenation-state of Mb alters the binding of fatty acids. For example, freshly prepared Mb showed higher binding compared to the commercially available Mb and conversion of MbO_2_ to met-Mb reduced fatty acid binding ~60–70% [11]. More recently, ^1^H NMR measurements demonstrated that the 8-methyl propionate of the heme group in Fe (III) MbCN displays a selective perturbation upon addition of palmitate [12]. Notably, Fe (III) MbCN is used as an alternate model for physiological MbO_2_ to study the ligated MbO_2_ [13–15]. Further support comes from NMR studies from Shih *et al*. which have shown that fatty acid does not interact with deoxy-Mb whereas ligated states of Mb do have specific and non-specific interactions with palmitic acid [16]. Physiological support for the linkage between globins and fatty acids is derived from the analysis of Mb knockout mice [5, 7]. These knockout mice exhibit decreased fatty acid oxidation and a biochemical shift from fatty acid to glucose oxidation [5, 7]. Proteomic and gene analysis results suggested that enzymes of mitochondrial β-oxidation are downregulated and glucose transporters are upregulated [7].

Interestingly, evolutionary analysis suggests that Mb and the fatty acid-binding protein albumin arose from a common fragment of a primordial globin gene [17]. Indeed, a recently-discovered globin member, cytoglobin, has been shown to interact with oleic acid with a high binding affinity [18]. The lipid-induced transformation of cytoglobin to a disulfide-linked dimer is proposed to be involved in generating cell signaling lipid molecules under an oxidative environment [18]. In addition, other reports demonstrate that the *Escherichia coli* flavohemoglobin binds specifically to unsaturated and cyclopropanated fatty acids [19]. Taken together, globins and fatty acids are physiological binding partners, thus Mb might play a key functional role in cardiac and skeletal cell fuel partitioning.

Despite extensive research in the role(s) for Mb in fatty acid biology, there is general lack of understanding regarding the 3D nature of the binding pocket, coordinating residues, and conformational switches which may underlie structural interaction of fatty acids with Mb. In the present study, we implemented a computational pipeline including sequence analysis, molecular docking and molecular dynamics simulations to define the structural determinants of this interaction. Taken together, our descriptive and predictive models accord with published findings and support the idea that Mb acts as a transporter or sequestration site for cellular fatty acid when it is in the oxygenated state and releases fatty acid when Mb converts to deoxygenated state [12].

## Materials and Methods

### Binding Site Prediction

To predict the fatty acid binding region in Mb, we used a Position Specific Scoring Matrix (PSSM) based method described previously [20, 21]. We started by defining an initial PSSM library from the experimentally determined Fatty Acid Binding Protein (FABP) regions in 42 well-characterized lipid binding crystal structures collected from the protein databank [22–25]. This initial PSSM library was leveraged to search for more FABP regions using psi-blast and thus expanded to 1185 FABP-specific PSSM libraries. Further, we aligned human and horse Mb with this expanded FABP specific PSSM library. All the positive alignments were recorded and a raw score for every residue was calculated by the summation of the alignment scores at each position. This score was normalized by subtracting the average residue score of the target protein from the raw scores.

This scoring scheme allowed for calculation of a residue score that represents the occurrence of identical and similar residues from each query-PSSM alignment above threshold. Using Smith-Waterman algorithm with two parameter sets such as (BLOSUM62, GOP = 10, GEP = 0.5) and (BLOSUM 45, GOP = 11, GEP = 1), we generated alignments in the query sequence with profiles that were positive by ADA-BLAST [20, 21]. Then, raw scores for each residue were calculated by scoring a value = 2 for identities and value = 1 for positive substitutions from each alignment. These values were summed for all alignments at each position to obtain a total residue score. The scores were normalized using the series of equations shown below. Eq (1) finds highly conserved residues whose score is above the average residue score in the sequence. Eq (2) recalculates the average score of these residues as a representative score for each sequence. Eq (3) calculates the norms of average scores to reduce the effect of the protein chain length. These data can be used to obtain thresholds with positive and negative training sets.

$${NS}_{residue}={SC}_{raw}-({SUM}_{total\;score}/{LEN}_{query})$$ Eq (1)

$${ASC}_{query}={SUM}_{PNS}/{NR}_{residue}$$ Eq (2)

$${NORM}_{avg\;score}={AS}_{query}\;*\;100/{LEN}_{query}$$ Eq (3)

where,

NS_residue_ = the normalized score of a residue

SC_raw_ = the raw score of a residue

SUM_total score_ = the sum of total query score

LEN_query_ = the query length

ASC_query_ = the average query score

SUM_PNS_ = the sum of positive normalized scores

NR_residue_ = the number of residues with positive scores

NORM_avg score_ = the normalized average score

AS_query_ = the average query score

### Construction of the oxy-Mb model

Due to the unavailability of the horse (*Equus caballus*) oxy-Mb crystal structure, we modeled oxy-Mb from the horse deoxy-Mb crystal structure (PDB ID: 2V1K) [26]. We transferred the coordinates of the oxygen molecule from the sperm whale oxy-Mb (PDB ID: 1MBO) [27] and constructed the horse oxy-Mb model. To attain equilibrium and stability, MD simulations (described below) were performed on the oxy-Mb complex for 10 ns before using the model for docking studies.

### Molecular docking

The initial structure of deoxy-Mb used for both molecular docking and MD simulations was obtained from the protein databank (PDB ID: 2V1K) [26]. The palmitic acid structure was obtained from the 3D structure of recombinant human muscle fatty acid-binding protein (PDB ID: 2HMB) and oleic acid is obtained from liver bile acid-binding protein (PDB ID: 2FTB) [25, 28]. To perform docking, AutoDock 4.2 [4] was used to obtain the initial protein ligand complex necessary for MD simulation. For all the docking calculations, we applied Lamarckian genetic algorithm (LGA) specified in the AutoDock. All the torsional angles for both palmitic and oleic acid are held flexible, whereas for both the proteins (deoxy-Mb and oxy-Mb), we held the torsional angles rigid, except for the lysine residues (Lys45 & Lys63) leaving them flexible [30]. These flexible residues allow specific side chain rotation around torsional degrees of freedom which is used to explore the conformational space of the flexible ligand [4]. Polar hydrogen atoms were added for both the protein structures using AutoDock tools and later Kollman united atom partial charges were assigned. Based on the binding site predicted from the fatty acid PSSM library, the grid size is set to 70 X 70 X 70 points with grid spacing of 0.375 Å. The gridbox is centered by taking into consideration coverage of those amino acids that span the high positional score region from sequence analysis. Maximum energy evaluations of 25,000,000 steps were performed with a population size of 300 while the total independent runs were fixed to 150. We used a clustering algorithm described in ADT/AutoDock [4], to group the similar conformation or “clusters” based on their lowest energy conformations and their RMSD to one another. In cases where AutoDock clusters docked results at 2 Å RMSD and the positions differed by less than 2 Å, these were taken as identical and represented by the energetically top ranked structures, as the energy differences within between the docked structures placed in the same cluster are generally small under these assumptions.

### Molecular dynamics simulation

To perform the MD simulations on the predicted protein-ligand complex, we used the NAMD package [31]. Simulation cell assembly, visualization and analysis of the results were performed using custom Tcl scripts in the VMD v1.9.1 [32]. For both the oxy-Mb and deoxy-Mb, default parameters from CHARMM27 force field for heme and oxygen were used. The required patches were applied to build the protein-heme-ligand complexes. In the case of the oxy-Mb it creates the proper charge distribution on the oxygen molecule and bonding between oxygen molecule, heme iron, and proximal histidine. All the MD simulations were performed using the NPT ensemble using CHARMM27 force field parameters [33, 34]. For oxygen-bound myoglobin, updated partial atomic charges of the heme prosthetic group and oxygen molecule were taken from Daigle *et al*. study, where the parameters were optimized using standard *Ab initio* quantum mechanical (QM) calculations [35]. Under these conditions, oxy-Mb was solvated with TIP3P water model [36] in a rectangular 3D periodic box of which the dimensions in every direction were chosen to be at least 10 Å larger than the solute. To maintain the electroneutrality of the whole system, a total of 26 Na^+^ & 25 Cl^-^ ions were added up to equivalent of 150 mM salt concentration in oxy-Mb. Constant pressure (1 atm) and temperature regulation (1K to 300K) with a collision frequency of 1.0 is achieved by Langevin Dynamics [37, 38]. Periodic boundary settings were maintained with the cutoff distance applied for non-bonded interactions is taken as 12 Å and particle mesh Ewald (PME) method is used to treat long-range electrostatic interactions with the switching distance 1.5 Å less than the cutoff. To avoid conflicting contacts, energy minimization steps were performed on the solvent (keeping the lipid-protein complex fixed) using the steepest descent in the first 3000 steps and then a conjugate gradient method in the subsequent 3000 steps. To attain equilibrium, the system is subjected to gradual heating until it reaches 300K at 1 atm. During the entire MD simulations, the coordinates of each system are saved for every 1 picosecond (ps).

## Results and Discussion

### Sequence-Profile Comparisons Predict Binding Pocket in Mb for Fatty acids

Since the fatty acid-binding pocket in Mb is unknown, we sought to computationally predict the ligand-binding site. There are several methods that may be employed, including Q-site Finder [39], SiteHound-web [40], COACH [41], BioLip [42], and FunFOLD2 [43]. Despite their utility, these methods are challenging to apply to predictions in which the comparator proteins are structurally dissimilar and exhibit high sequence divergence. For instance, Mb and Fatty Acid Binding Protein (FABP) have many structural differences. Mb has an eight alpha helical structure while FABP family proteins are 10-stranded anti-parallel beta-barrels. Thus, identifying shared functional motifs through direct structural superposition is difficult.

We reasoned that sequence-profile comparisons in Mb with a reference library of fatty acid-binding sequences might successfully isolate subtle, yet shared sequence features, which are not easily identified by sequence-sequence or structure-structure comparisons. Hence, we generated a position specific scoring matrix (PSSM) library constructed from fatty acid-binding regions of 1185 FABP protein family sequence profiles (see Methods for complete description). In similar implementations, these PHYRN-based PSSM libraries delivered high resolution phylogenies in highly divergent datasets (~7% sequence identity) and identified functional residues involved in protein binding and interactions sites [44–46].

**Fig 1:**
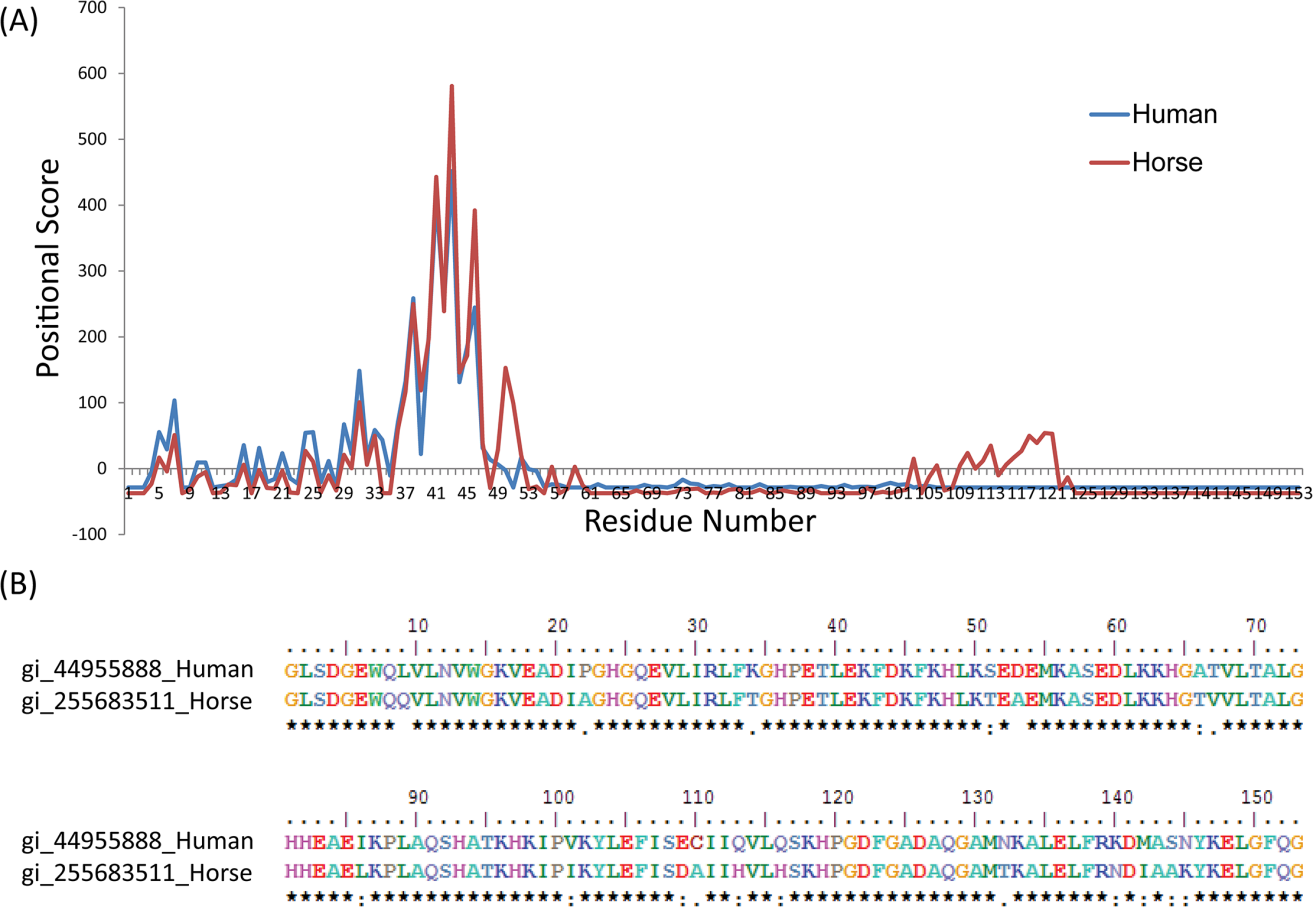
(A) Positional score results of both horse and human myoglobin amino acid sequences scored against the Fatty Acid Binding protein (FABP) PSSM library. A high peak is observed in the region spanning from amino acid positions 31 to 50. (B) Pairwise alignment of horse and human sequences revealed the highest positional score is assigned to the residue Lys45.

The crystal structures of the reference FABPs are ~110–160 amino acids in length and share a common fold around an internal hydrophobic cavity where the fatty acid is bound, mainly through the side chains of the amino acids arginine, lysine and glutamine residues through hydrogen bonding and electrostatic interactions [25]. Comparatively, Mb also has a hydrophobic core in the center where the heme is held through the proximal histidine group directly to the iron center. Using our FABP-specific PSSM library, we screened both horse and human Mb sequences to assess any shared sequence features which may indicate putative fatty acid-binding residues. As shown in the **Fig 1A**, horse and human sequences show a common, enriched positional score spanning the region from residues 31 to 50. The highest positional score identified the residue Lys45, which may indicate a direct role in fatty acid binding (see **Fig 1B**). Overlaying these scores on the 3D Mb reference structure reveals that all the high scoring residues predominate next to the heme group of Mb. This putative pocket for the fatty acid is highly feasible considering the hydrophobic core and the NMR studies showing selective perturbation of heme upon addition of palmitate [12].

### Molecular Docking Reveals Distinction Between oxy-Mb and deoxy-Mb

**Fig 2:**
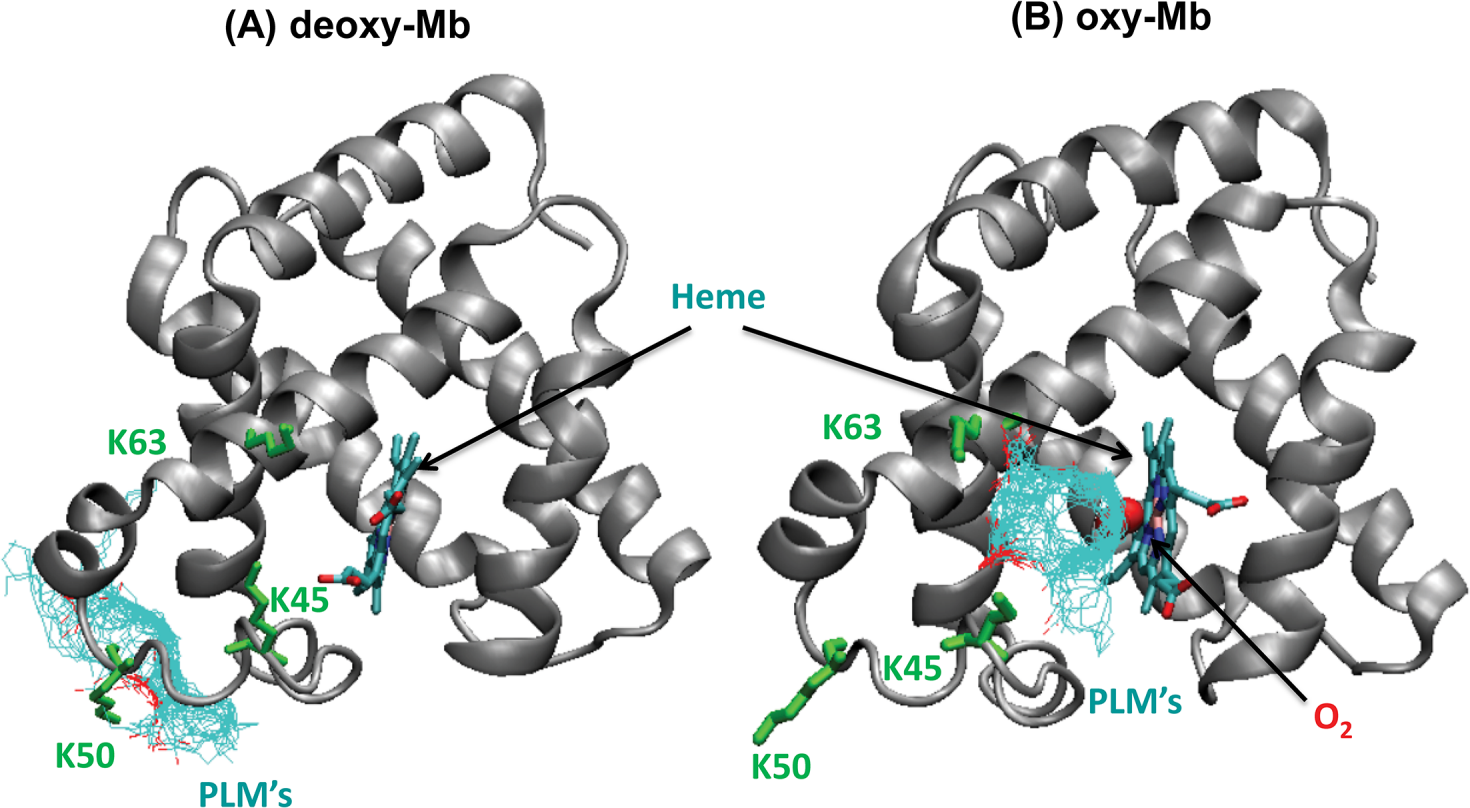
Cluster analysis from AutoDock displaying docking conformations of (A) palmitic acid (PLM) with horse deoxy-Mb and (B) PLM with oxy-Mb (the first 50 models for PLM are displayed for clarity, with each model’s PLM depicted as individual light blue lines).

We implemented the AutoDock program with the reference Mb structures with a gridbox centered on those amino acids that span the high positional score region from the sequence-comparisons above. Notably, AutoDock predicted significant differences in the docking position of palmitic acid with deoxy-Mb versus oxy-Mb. The best docking solutions of palmitic acid with deoxy-Mb and oxy-Mb, along with cluster results, are shown in **Fig 2A and 2B**, respectively. The analysis predicts that 90% of the clusters in the docking results of deoxy-Mb bound palmitic acid to Lys50, which is located away from the porphyrin group, with a predicted binding energy of -4.12 kcal/mol (see **Fig 2A)**. Comparatively, in the oxy-Mb structure, 83% of the clusters of palmitic acid bound in the hydrophobic cleft near the heme group, which involves both Lys45 and Lys63 residues with a binding energy of -5.99 kcal/mol (see **Fig 2B)**. Similar cluster results were observed in the docking predictions of oleic acid with oxy-Mb structure with an estimated binding free energy of -6.09 kcal/mol. Even though the difference in the binding energies between the palmitic and oleic acid are smaller, the calculated values are not the absolute binding energies, but just representative measures of how favorably the ligand binds to the protein [29]. In the oxy-Mb structure, some of the binding modes of carboxyl group of both palmitic and oleic acid were seen to interact with amino group of Lys63. We were encouraged by the initial binding modes of both the fatty acids with oxy-Mb considering that the ligand tail is in close proximity with the 8-methyl propionate of heme, shown to be the basis of signal in the NMR studies in the titration of palmitate with Mb [12].

### Molecular Dynamic Simulations of the Fatty-Acid Binding Pocket

**Fig 3:**
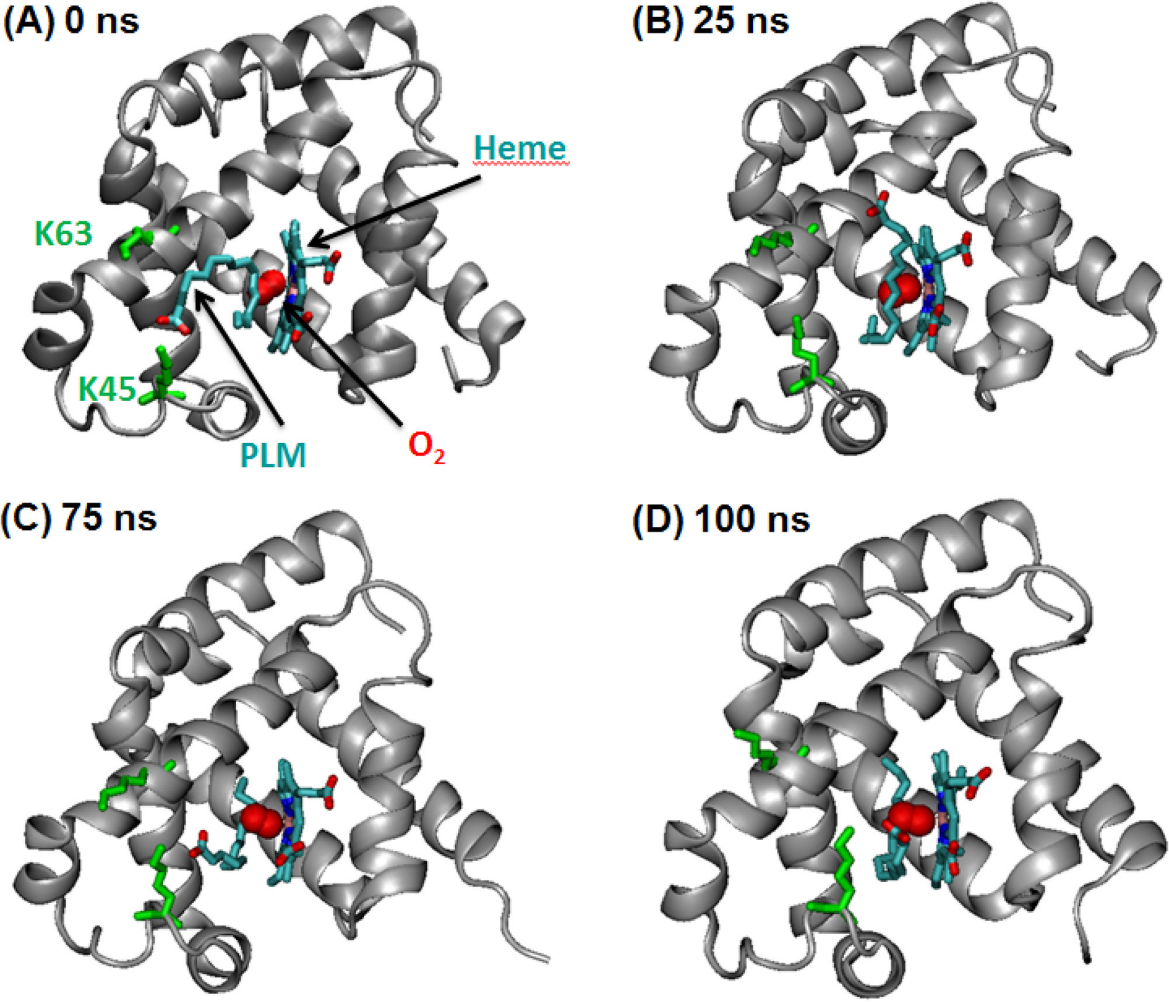
Snapshots of horse oxy-Mb with palmitic acid (PLM) system configuration during 100 nanosecond (ns) simulation with intervals of (A) 0 ns, (B) 25 ns, (C) 75 ns, and (D) 100 ns. The protein backbone is represented as a cartoon, whereas PLM, heme, Lys45 & Lys63 (colored green) are displayed as sticks and the oxygen molecule is represented in a ball shape (colored red). Water molecules are excluded for clarity.

In order to gain insights into the structural dynamics and stability of both the palmitic acid-Mb and oleic acid-Mb interactions, we selected the top docked conformations from the AutoDock results, and performed an extended 100 ns molecular dynamic simulation study to optimize the oxy-Mb and ligand complex (see **Fig 3**). During the 100 ns MD simulation run, the head carboxyl group of both palmitic and oleic acid interacts with the amino group of Lys45, and adjacently Lys63 and His64, in the hydrophobic groove next to the heme region. This region stays stably dehydrated, suggesting that the arrangement is stable. Primarily, both ligands are docked in a “U” shaped conformation, where the head carboxyl group makes polar contacts with the positive amino group of Lys45 and Lys63, whereas the tail (e.g. alkyl group from C10 to C16 of palmitic acid) is facing toward the heme group (see **Fig 3A**). Importantly, the observed “U” shaped conformation of fatty acids in the Mb pocket is a characteristic fatty acid conformation in the binding site of known FABPs [47].

**Fig 4:**
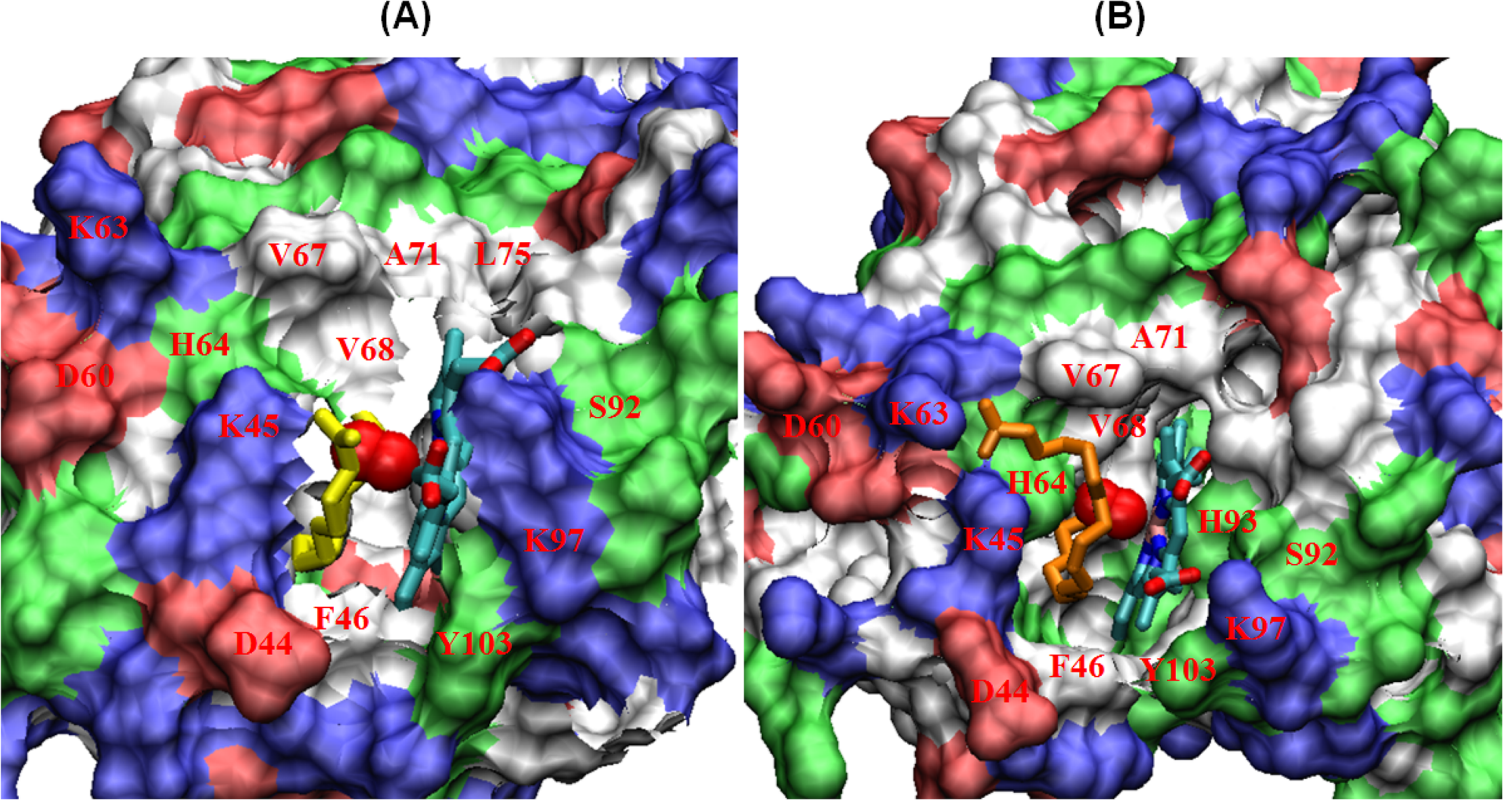
(A-B) Surface representation of both palmitic acid (PLM) and oleic acid (OLE) binding to horse Mb. The binding site occupied with a ligand molecule highlights the surrounding hydrophobic residues and the charged residues on the end anchors of the PLM head group. Water molecules are excluded for clarity. PLM (yellow), OLE (orange) and heme are displayed as sticks, whereas oxygen molecule are represented as balls.

Initially, in the oxy-Mb and palmitic acid complex, the alkyl tail spontaneously enters deeper inside the hydrophobic cleft while reducing the exposure of the tail to water, where palmitic acid is stabilized by the hydrophobic residues Leu29, Leu32, Phe33, Phe43, Phe46, Val67, Val68 and Ile107 (see **Fig 3B–3D**). Phe33, Phe43 and Phe46, which are inside the hydrophobic groove, are displaced slightly, resulting in the gate opening for the alkyl tail of palmitic acid, whereas Leu29 and Phe46 are found to interact with the alkyl tail of palmitic acid (see **Fig 4A**). Notably, due to the absence of a double bond in palmitic acid, the alkyl tail movement is not hindered, moves swiftly and occasionally attains a linear shape structure during the 100 ns simulation. Oleic acid exhibits a similar pattern in binding position when compared to palmitic acid. Oleic acid starts as a “U” shaped structure and during the simulation, the alkyl tail spontaneously enters the hydrophobic pocket maintaining the “U” shape structure (see **Fig 4A and 4B** and also **S1** and **S2 Files (video)**). Due to the presence of cis double bond between C9 and C10, the movement of the alkyl tail is limited. Therefore, it does not favor free rotation which also helps the hydrophobic tail in attaining a “U” shaped structure into the hydrophobic pocket of oxy-Mb. The fatty acid tail of oleic acid also makes similar contacts with surrounding hydrophobic residues Leu29, Phe33, Phe43, Phe46, Val67, Val68 and Ile107 (see **Fig 4B**).

Analyzing the entire simulation run for both the fatty acids, we observed that not all protein contacts are retained continuously over time. The movement of both palmitic acid and oleic acid are in and out of the pocket to varying degrees. Comparing the average RMSF (Root Mean Square Fluctuation) trajectories for the two fatty acids over 100 ns, oleic acid appears more stable as it exhibits smaller movement when compared to the palmitic acid (**S1 Fig**). During this process, palmitic acid makes additional contacts with residues Val28, Leu69, Leu72 and Ile111. The carboxyl head group in both fatty acids exhibited hydrogen-bonding interactions with Lys45 and Lys63. The results show that these head groups switch between Lys45 and Lys63 during the 100 ns simulation, and do not stick to one place. Across the 100 ns simulation, palmitic acid molecule exhibited H-bonding with Lys45 and Lys63 22.4% and 2.3% of the time respectively, whereas in oleic acid H-Bonding for Lys45 and Lys63 are predicted as 38.32% and 11.78% of the time respectively. Occasionally, the carboxyl head of both the fatty acids are also involved in the hydrogen bonding interactions with the surrounding water molecules.

The distance penetrated by the alkyl tail of palmitic acid and oleic acid (distance measured by the position of last carbon) starting from the first frame to the last frame of the total 100 ns simulation run was measured to be 6.92 Å and 5.98 Å respectively. Both these results indicate that the fatty acids are not trapped behind some structural barrier just by our initial placement of it in the simulation model, as they readily move around the binding site but regain the contact while remaining within the pocket. This supports the idea that this binding site is indeed the energy minimum. This dynamic behavior also shows that the fatty acid is in principle capable of exchange with the bulk, which agrees with the proposed fatty-acid carrier function of Mb. It should be noted that existence of the entrance/exit pathway without huge prohibitive barriers did not immediately follow from the docking studies and was revealed only by MD simulations. This infers that the binding is not excessively tight, and the energy minimum might be shallow enough for fatty acids to leave the binding site when oxygen is released. This agrees with the reasonable scale of binding energy predicted from the docking studies and also accords with experimental finding of low affinity binding [10, 12]. During the entire 100 ns simulation run, the fatty acids do not show any direct interaction with the bound oxygen molecule, instead the alkyl tail interacts with the hydrophobic residues in the vicinity of the heme, while the head carboxyl group of fatty acid is stabilized with the amino group of both Lys45 and Lys63.

Control simulations assessed the binding of palmitic acid to oxy-Mb structure in two different ways. In the first method, we mutated both the lysine residues (K45A & K63A) and performed the docking studies using AutoDock. The results are similar to the docking studies with deoxy-Mb, in which all the palmitic acid is bound to Lys50 and does not bind near the heme region (see **Fig 2A**). In the second method, the starting docking complex of oxy-Mb with palmitic acid was taken, and subsequently we mutated both the lysine residues (K45A and K63A). In addition, we also removed the oxygen molecule and ran the MD simulations. Under these conditions, in the first 10 ns MD simulation run, the ligand exits out from the binding site due to the absence of coordinating lysine residues and the oxygen molecule which is involved in stabilizing the alkyl tail of palmitic acid in the hydrophobic cleft (see **S3 File—(video)** and **S2 Fig**).

**Fig 5:**
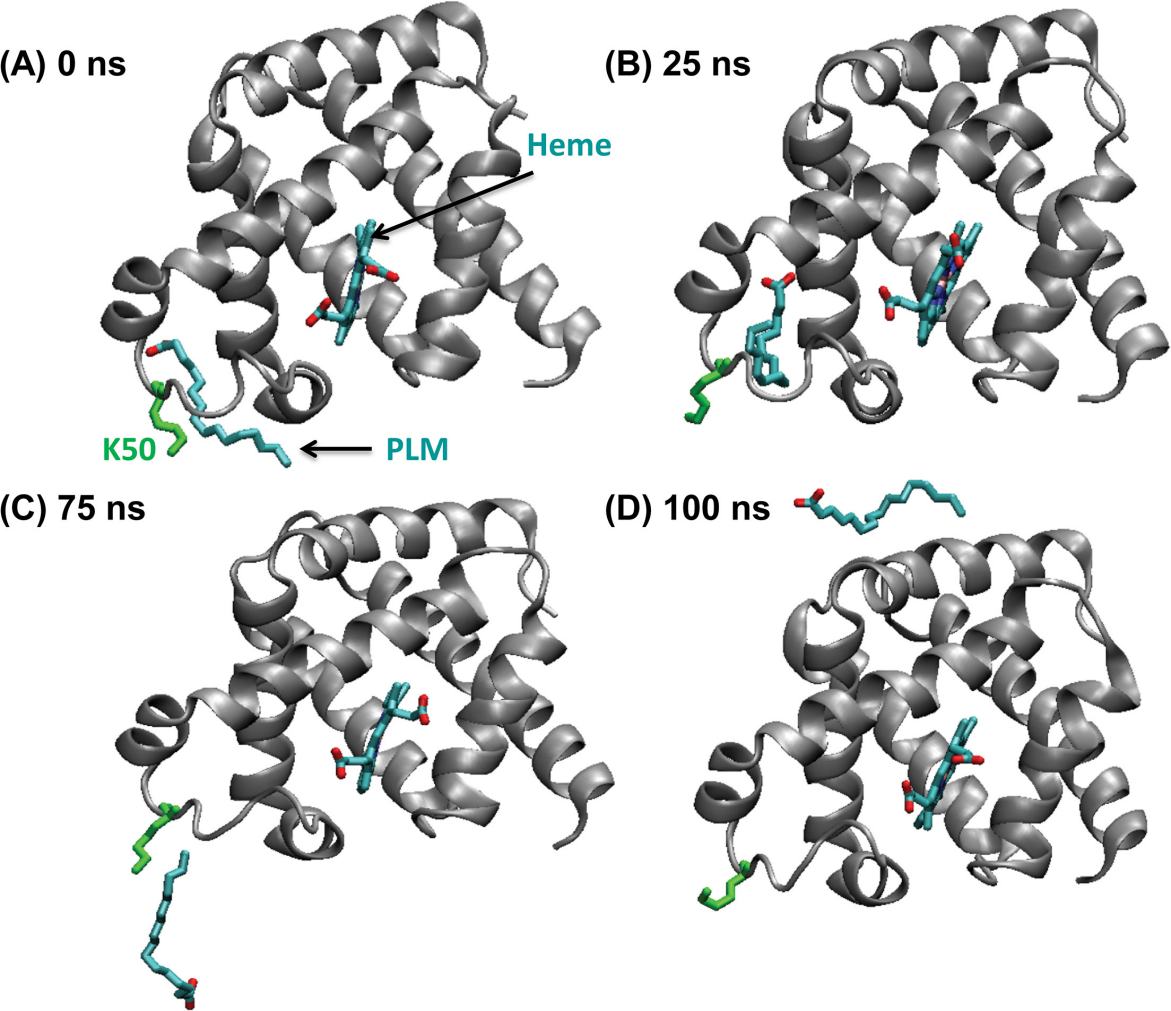
Snapshots of horse deoxy-Mb with palmitic acid (PLM) system configuration during 100 ns simulation with intervals of (A) 0 ns, (B) 25 ns, (C) 75 ns, and (D) 100 ns. The protein backbone is represented as a cartoon, whereas PLM, heme and Lys50 are displayed as sticks. Water molecules are excluded for clarity.

Additional 100 ns MD simulations were performed on the best docked structure of deoxy-Mb with palmitic acid ligand. Under these no-oxygen conditions, initially palmitic acid is docked to the Lys50, but the interaction is not stable and with time moves away when exposed to the hydrophilic environment (see **Fig 5** and **S4 File (video)**). To add more statistical weight to our conclusions, we have conducted four extra 20 ns MD simulations on the deoxy-Mb fatty acid complex. In these runs palmitic acid is placed at 4 different starting points, to check whether the interaction is stable. For the first three starting points (**S3A–S3C Fig)**, we have chosen alternate binding poses (the next best energetically favorable binding poses) predicted by the Autodock using the same grid box dimensions, where palmitic acid is bound near Lys47, Lys62 or Lys50 respectively. To test for the alternate binding site, we also placed palmitic acid manually (not predicted by the Autodock) at Lys102 where there is a small opening for the hydrophobic grove, to test whether fatty acid would enter through the other end of the oxy-Mb (**S3D Fig**). Out of the four MD runs, three of the results (**S3A, S3B and S3D Fig)** match our previous result of deoxy-Mb fatty acid complex where fatty acid escapes into the hydrophilic environment. In the fourth run where the fatty acid is placed near the Lys62 (**S3C Fig**) close to the binding residue Lys63, shows weak interaction where it first loses its contact with the amino group of Lys62 and during the MD run, it regains its contact with the amino group of Lys63, but the tail is left exposed to the hydrophilic environment, unable to penetrate into the binding pocket near heme.

The observed binding of palmitic acid to oxy-Mb, and not deoxy-Mb, might be explained by the conformational change that happens at the heme-His linkage upon oxygenation. X-ray crystallographic studies on Mb revealed that, upon oxygenation, heme becomes slightly planar from the usual domed structure. This results in a slight decrease in the coordination bond length between the Fe and histidine (N^€2^ [epsilon nitrogen of His93] and the imidazole ring) which takes a more symmetric position with respect to the heme due to the degree of heme tilting [27, 48]. In stand-alone oxy-Mb, the residues Lys45 and Val67 contact the polar atoms of the heme, while the insertion of palmitic acid intercepts these residues and modestly deforms the heme, pushing it away from the usual position. This dynamic change of heme could result in a change of its spectral and dynamic properties, which is in accordance with experimental results of Sriram et al. [12]. Based on the present MD simulations, we propose a fatty acid entry mechanism for oxy-Mb. When the Mb is in the oxygenated state, the fatty acid binding site is more opened by the conformational change of heme, allowing access to part of the outer binding site that has a positive charge in the form of lysines. Further, the fatty acid is pushed by water inside the hydrophobic grove with its tail-in orientation gaining the contact with the surrounding hydrophobic residues which helps in the passage of both the fatty acids. The fatty acid thus entered allows the head group to be accommodated near the positively charged Lys45 and Lys63 binding pocket.

In oxy-Mb, His93 (proximal) is the key residue involved in holding the heme molecule that positions the iron to coordinate with the O_2_ molecule. Conversely, His64 (distal) is involved in the reversible binding of oxygen and is not in direct contact with the iron. Further, His64 forms a hydrogen bond with the second atom of the O_2_ molecule forming a Fe-O-O-His64 complex. However, it has been suggested that the swinging motion of the imidazole ring of His64 regulates the rate of O_2_ entry in the globins [49]. Additionally, experimental evidences in mammalian myoglobin and hemoglobin have shown that His64 acts as a ‘gate’ for ligands in both entry and exit pathways [50]. Unlike the proposed mechanism in which the open and closed ‘gate’ conformations regulate O_2_ uptake, recent simulation studies predict that O_2_ uptake is due to the increase in hydrophobicity near the O_2_ binding pocket which is formed by the surrounding residues Ile28, Leu29, Leu32, Val68 and Ile107 [51].

In the present computational studies, fatty acid binding to oxy-Mb revealed a closed ‘gate’ conformation, indicating that fatty acid binding restricts the mobility of His64 side chain. Our MD simulations also suggest a mechanism for fatty acids potentially regulating the release of bound O_2_ where the amino acids Leu29 and Phe46 (residues which interact with alkyl tail of fatty acids) were also found to limit the mobility of His64. Future molecular and structural studies will advance our understanding of relationships between the presence of fatty acids, dynamics of His64 and binding of oxygen in the FA-oxy-Mb complex.

## Conclusion

In this study we gleaned mechanistic insight into the structural determinants of both palmitic acid and oleic acid binding with oxy-Mb. The sequence analysis and the docking results strongly suggest that these fatty acids bind near the hydrophobic region of the heme group. Extended MD simulations of the fatty acids with the oxy-Mb have shown that the head group of the fatty acid interacts with Lys45 and Lys63, whereas the tail is pushed deep inside the hydrophobic cleft surrounded by hydrophobic amino acids.

Importantly, the current MD simulations provide insight into the mechanism of how fatty acids bind to Mb only in the oxygenated state and do not show any interaction when Mb is in the deoxygenated state. Further studies, including binding and site-directed mutagenesis experiments will be required to validate the models through testing the simulation-derived predictions. For mutational analysis, the two interesting candidates for mutations would be the “gate-keeping” residues Phe46 and His64. These two residues define the water boundary at the entrance to the fatty acid-holding cavity and do not directly touch the heme (while fatty acid is present), but are in tight contact with the fatty acid. Also, these two residues Phe46 and His64 are on the alpha-helices, which makes the position of the backbone restrained, while the side chains are partially accessible from the outside and do not participate in a tight inter-helical packing. It is likely that the placement of a bulky tryptophan residue in these locations will sterically hinder the penetration of the fatty acid tail. Also, hydrophilic mutations (e.g. Phe46Gln or His64Gln) will probably make the binding unstable by replacing a relatively hydrophobic side chain with a polar residue with water cap (i.e. decrease in hydrophobic interactions). Similar effect might be expected if these positions are mutated to Ala as it has very small side chain.

Overall, the results presented here indicate that myoglobin serves as a potentially important transporter and sequestering site of long chain fatty acids in muscle and cardiomyocytes. Furthermore, the results suggest that tissue oxygenation has a major impact on the amounts of sequestered versus unbound long chain fatty acids associated with myoglobin, which could have important implications for modifying myocyte fuel partitioning during ischemia or intense exercise.

## Supporting Information

S1 Fig Root Mean Square Fluctuation (RMSF) of palmitic and oleic acid as a function of time when bound to oxy-Mb protein showing the deviations by residue. Only heavy atoms are taken into consideration while calculating RMSF (hydrogens were excluded). Numbers of residues in each molecule along with the oxygen atoms are 18 and 20 for palmitic and oleic acid respectively
(TIF)

S2 Fig Snapshots of the mutated horse oxy-Mb and palmitic acid (PLM) complex where oxygen is removed and both the lysines are mutated to alanines (K45A and K63A); system configuration during the initial 10 ns simulation with intervals of (A) 0 ns, (B) 2.5 ns, (C) 5 ns, and (D) 100 ns. The protein backbone is represented as a cartoon, whereas PLM, heme, Ala45 and Ala63 are displayed as sticks.
(TIF)

S3 Fig MD simulations of deoxy-Mb with palmitic acid (PLM) structure with four different starting points. The protein backbone is represented as cartoon shape, whereas heme and Lysines are displayed as sticks. (A) PLM is placed near Lys47, (B) PLM is placed near Lys62, (C) PLM is placed near Lys50, and (D) PLM is placed near Lys102. The starting representation of PLM in each of the four different runs is displayed as sticks (colored orange). The representative orange lines shows its movement during its 20 ns MD run, whereas the final frame of the PLM is indicated as sticks (colored green).
(TIF)

S1 File A compressed video of 100 ns simulation of oxy-Mb with palmitic acid (PLM). The protein backbone is represented as cartoon and colored as residue type, whereas PLM (colored yellow), heme, His64 are displayed as sticks. Both Lys45 and Lys63 are also displayed as sticks (colored blue), whereas oxygen molecule is represented in a ball shape (colored red). Water molecules are excluded for clarity.
(AVI)

S2 File A compressed video of 100 ns simulation of oxy-Mb with oleic acid (OLE). The protein backbone is represented as cartoon and colored as residue type, whereas OLE (colored orange), heme, His64 are displayed as sticks. The black color line in the oleic acid represents the double bond. Both Lys45 and Lys63 are also displayed as sticks (colored blue), whereas oxygen molecule is represented in a ball shape (colored red). Water molecules are excluded for clarity.
(AVI)

S3 File A compressed video of 10 ns simulation of mutated oxy-Mb with palmitic acid (PLM) where oxygen is removed and both the lysines are mutated to alanines (K45A and K63A). The protein backbone is represented as a cartoon, whereas PLM, heme, Ala45 and Ala63 are displayed as sticks. Water molecules are excluded for clarity.
(MPG)

S4 File A compressed video of 100 ns simulation of deoxy-Mb with palmitic acid (PLM). The protein backbone is represented as a cartoon, whereas PLM, heme, Lys50 (green) are displayed as sticks, whereas oxygen molecule is represented in a ball shape (colored red). Water molecules are excluded for clarity.
(MPG)
