# Supplementary figures and images for: Molecular Dynamic Simulations Reveal the Structural Determinants of Fatty Acid Binding to Oxy-Myoglobin
Source: PLoS One. 2015 Jun 1;10(6):e0128496. doi: 10.1371/journal.pone.0128496 (PMC4451517; doi:10.1371/journal.pone.0128496)

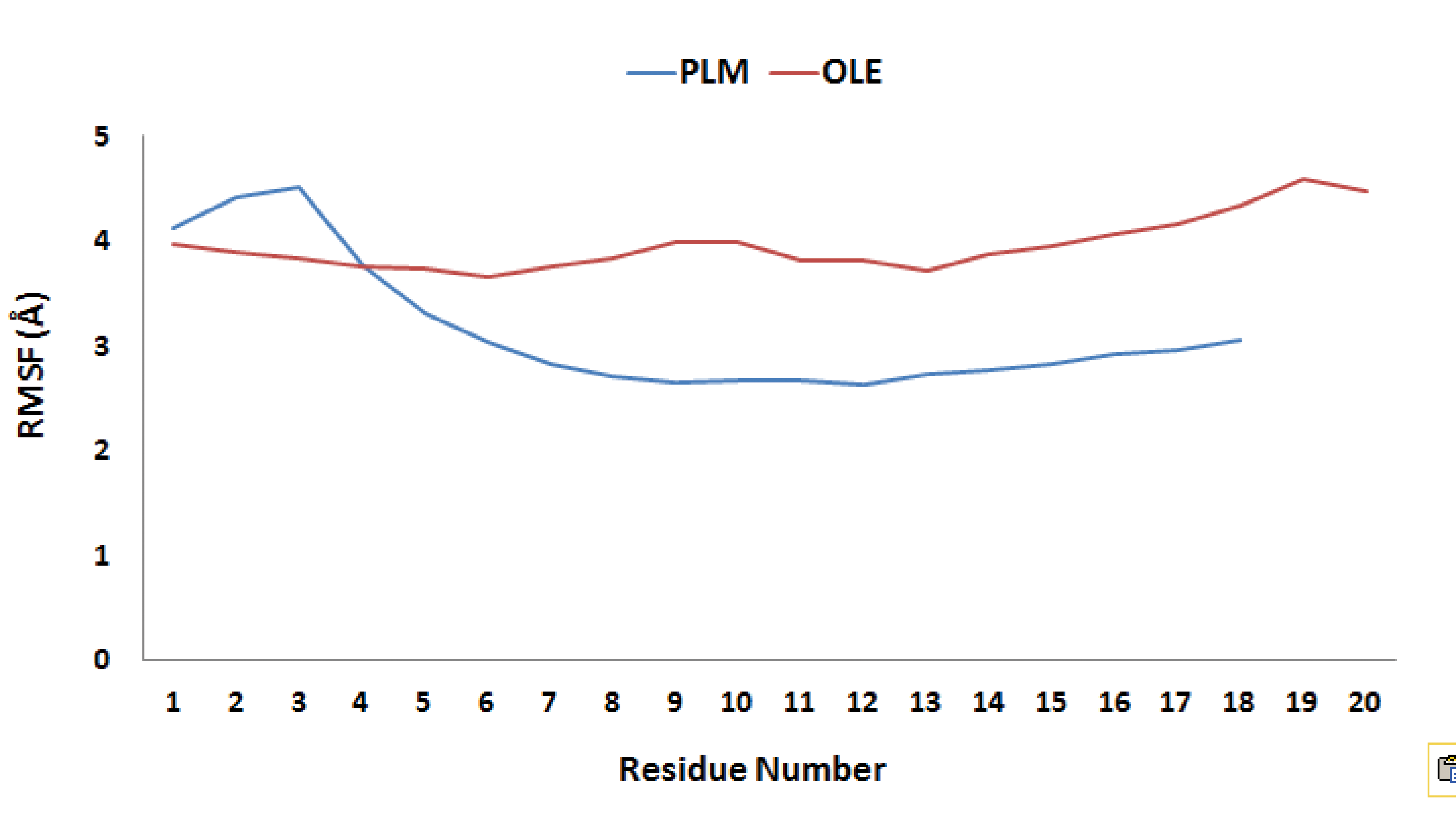

Supplement: S1 Fig — Only heavy atoms are taken into consideration while calculating RMSF (hydrogens were excluded). Numbers of residues in each molecule along with the oxygen atoms are 18 and 20 for palmitic and oleic acid respectively (TIF) [file pone.0128496.s001.tif]

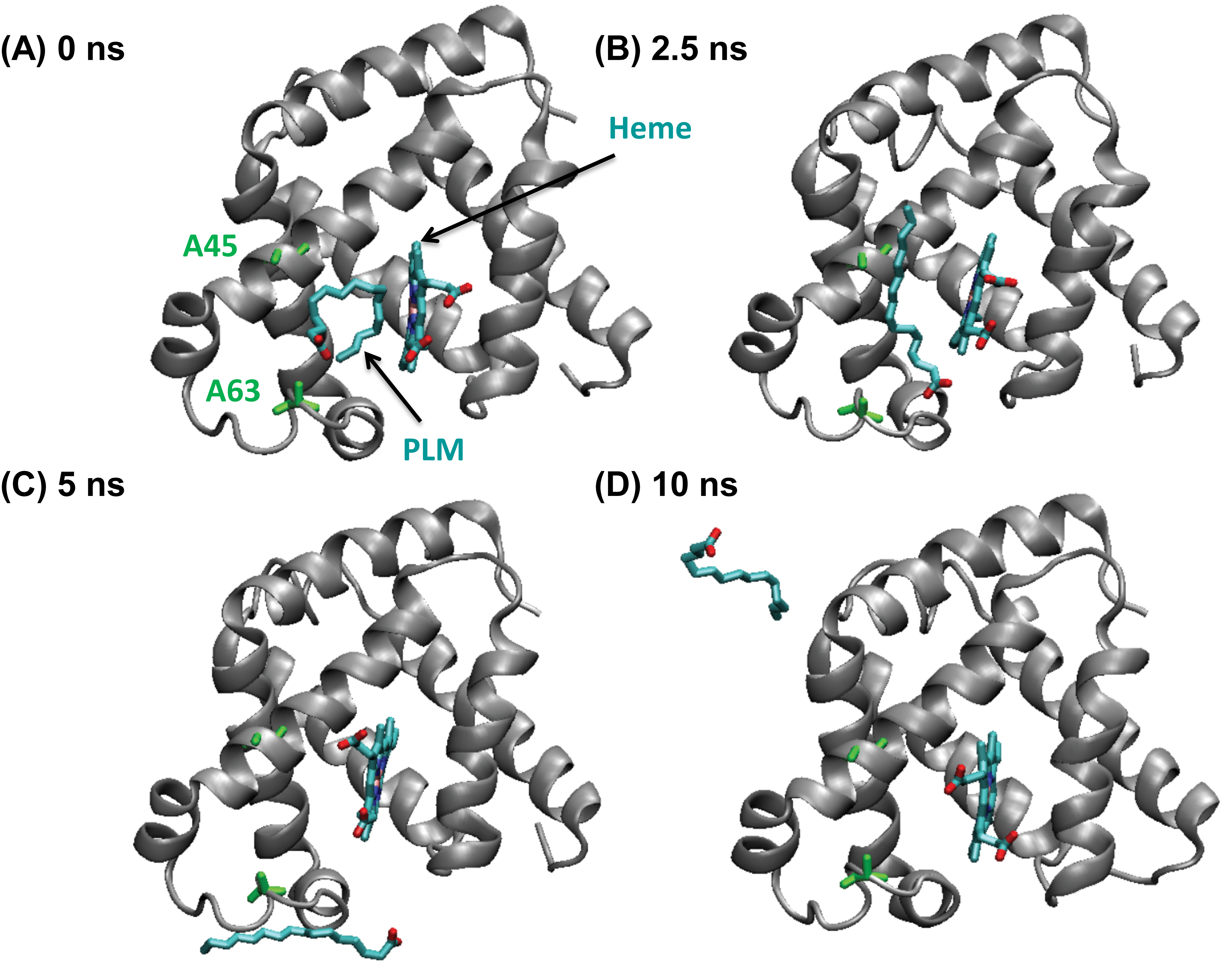

Supplement: S2 Fig — The protein backbone is represented as a cartoon, whereas PLM, heme, Ala45 and Ala63 are displayed as sticks. (TIF) [file pone.0128496.s002.tif]

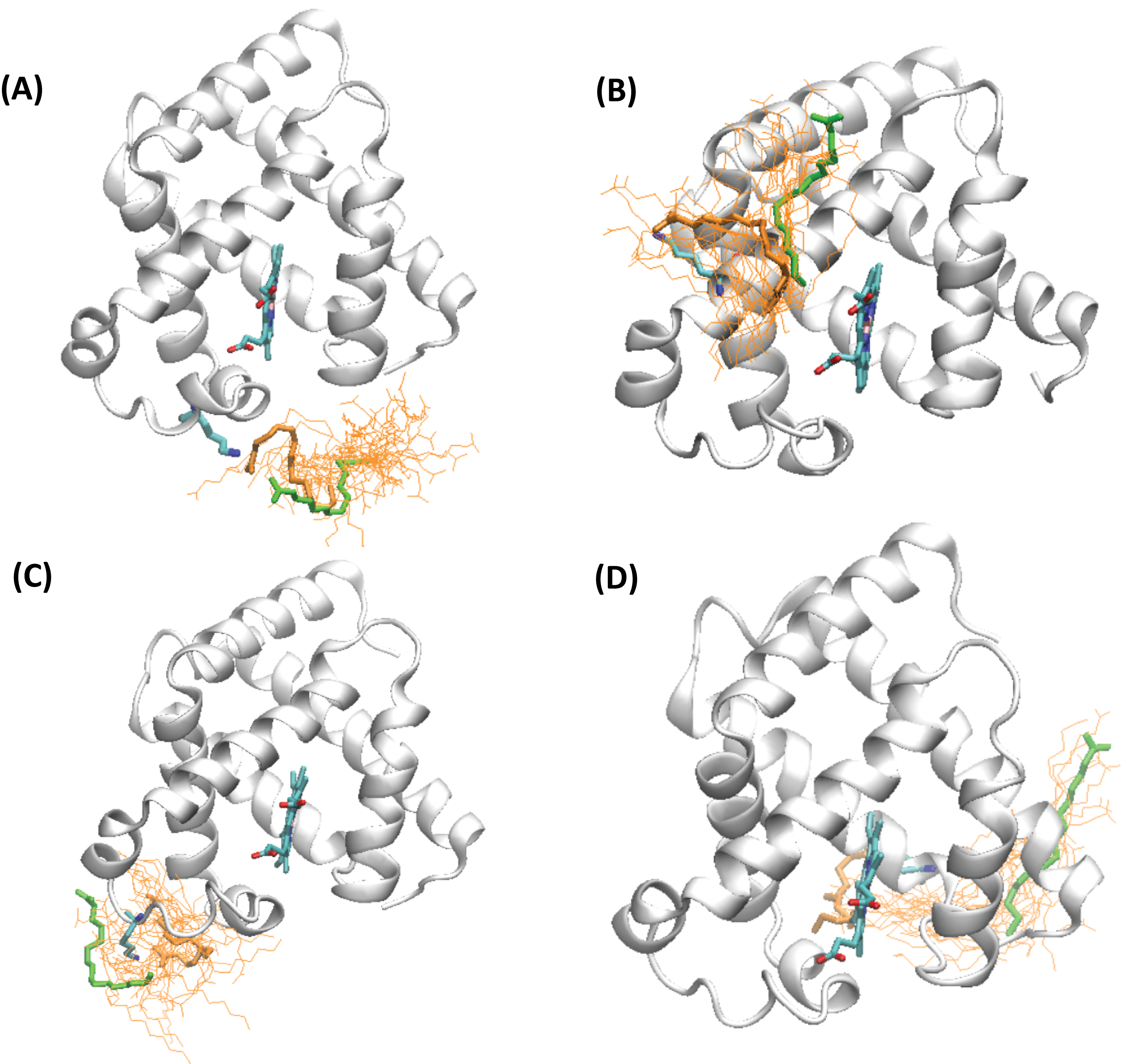

Supplement: S3 Fig — The protein backbone is represented as cartoon shape, whereas heme and Lysines are displayed as sticks. (A) PLM is placed near Lys47, (B) PLM is placed near Lys62, (C) PLM is placed near Lys50, and (D) PLM is placed near Lys102. The starting representation of PLM in each of the four different runs is displayed as sticks (colored orange). The representative orange lines shows its movement during its 20 ns MD run, whereas the final frame of the PLM is indicated as sticks (colored green). (TIF) [file pone.0128496.s003.tif]
